# Studies on the Content of Selected Technology Critical Elements (Germanium, Tellurium and Thallium) in Electronic Waste

**DOI:** 10.3390/ma14133722

**Published:** 2021-07-02

**Authors:** Joanna Willner, Agnieszka Fornalczyk, Magdalena Jablonska-Czapla, Katarzyna Grygoyc, Marzena Rachwal

**Affiliations:** 1 Faculty of Materials Engineering, Silesian University of Technology, 40-019 Katowice, Poland; Joanna.Willner@polsl.pl; 2 Institute of Environmental Engineering, Polish Academy of Sciences, 41-819 Zabrze, Poland; magdalena.czapla@ipispan.edu.pl (M.J.-C.); katarzyna.grygoyc@ipispan.edu.pl (K.G.); marzena.rachwal@ipispan.edu.pl (M.R.)

**Keywords:** technology-critical element (TCEs), e-waste processing, waste electrical, electronic equipment (WEEE)

## Abstract

The article draws attention to the problem of the presence of metals: germanium (Ge), tellurium (Te), thallium (Tl), and others (Cd, Ba, Co, Mn, Cr, Cu, Ni, Pb, Sr, and Zn) in selected waste of electrical and electronic equipment (WEEE). As a result of the growing demand for new technologies, the global consumption of TECs has also been increasing. Thus, the amount of metals in circulation, of which the impacts on the environment have not yet been fully understood, is constantly increasing. Due to the low content of these metals in WEEE, they are usually ignored during e-waste analyses. The main aim of this study was to determine the distribution of Ge, Te, and Tl (and other elements) in ground sieve fractions (1.0, 0.5, 0.2, and 0.1 mm) of selected electronic components (solar lamps, solar cell, LED TV screens, LCD screens, photoresistors, photodiodes, phototransistors) and to determine the possible tendency of the concentrations of these metals in fractions. This problem is particularly important because WEEE recycling processes (crushing, grinding, and even collection and transport operations) can lead to dispersion and migration of TCE pollutants into the environment. The quantitative composition of e-waste was identified and confirmed by ICP-MS, ICP-OES and SEM-EDS, and XRD analyses. It was found that Ge, Te, and Tl are concentrated in the finest fractions of ground e-waste, together with Cd and Cr, which may favor the migration of these pollutants in the form of dust during storage and processing of e-waste.

## 1. Introduction

Germanium and tellurium are technology-critical elements (TCEs) in the European Union (European COST Action TD1407: Network on Technology-Critical Elements) [1] and are crucialfor strategic sectors and new technologies, such as renewable energy, electric mobility, defense, aerospace, and digital technologies. Thallium, despite its high toxicity, is used in the electronics industry as well. Nowadays, these three metals are widespread in the waste of electrical and electronic equipment (WEEE), and, at the same time, are neglected and little known. Therefore, more attention should be paid to these metals present in electronic waste, not only due to possibility of their recovery, but also as they can be source of environment pollution, releasedd from WEEE recycling sites. The first stage of the recycling chain, including collection and processing of e-waste, e.g., shredding, cutting, and grinding, especially in open air, can be a source of uncontrolled emissions of dust pollutants containing metals and other organic and inorganic compounds. Transported over some distance, dust can easily migrate to the immediate surroundings of e-waste collection plants (or longer distances depending on particle sizes), penetrating into water and soil environments.

The possible presence of TCE in the environment and their impact on environmental processes should be particularly taken into account, especially if they constitute potentially toxic compounds [2]. Previous works of Sepúlveda et al. [3] and Uchida et al. [4] confirmed that dust and fine particles generated by the dismantling and sorting of e-waste are one of the pathways of soil contamination with metals (Pb, Ca, Cu, Mo, Ni, Sn, and Zn). Characterization of the source of e-waste, being the carrier of Ge, Te, Tl, requires knowledge of the quantitative and qualitative identification and recognition of the distribution of these elements in the fraction of ground e-waste material for further analysis of the issues related to the anthropogenic impact of TCE on the environment in areas surrounding e-waste collection and recycling plants.

## 2. Characteristics of TCEs and Their Impact on the Environment

Germanium is an element that is often used in industry because of its semi-metallic nature as well as its semiconductor properties. The alloy of this metal, along with small admixtures of arsenic, gallium, indium, antimony, or phosphorus, is used to build transistors, which are essential components in electronic devices. Germanium and its GeO_2_ oxide are transparent to infrared radiation; therefore, they are used as lenses and windows in optical instruments for the appropriate spectral range, and are used to detect thermal objects (this accounts for 30% of Ge applications). About 20% of produced germanium is used in optical materials (optical fibers) and in catalysts for the production of polyesters (polyethylene terephthalates, PET), as well as for synthetic textile fibers and photographic film [5,6].

The applications of tellurium are very diverse, but the quantities consumed are minimal, much smaller than many precious metals and semi-metals used in electronics. Initially, tellurium was used with lead as an alloying addition to steel. It was also used in catalysts, alloys with copper and lead (addition of tellurium to non-ferrous metals improves physical properties and resistance to chemical corrosion), for vulcanization of rubber, and in photocopiers and thermo-electronic equipment. Currently, tellurium is mainly used in the production of cadmium telluride (CdTe) thin-film solar cells, which is its major application (40% of global consumption), followed by thermo-electrics (30%). The cadmium present in cells would be toxic if released, however, this is impossible during normal operation of the cells. However, there is a risk of the release of harmful compounds during milling or other processes related to waste management [7]. In the case of tellurium, a majority of it is obtained as a by-product of non-ferrous metal-refining processes.

Thallium is used in semiconductor materials, photocells, infrared measuring devices, or in glass lenses, prisms, and windows for optical fibers; it is also used as a catalyst in organic synthesis. This element is important for the production of glasses with a high density and refractive index, optical lenses, imitation jewelry, electrochemical equipment, and corrosion-resistant alloys. Today, approximately 70% of thallium production is used in electronic devices [5]. Although thallium is a toxic element, it is used industrially and for the production of pesticides. In some countries, it has contributed to contamination—primarily of soil, water, and plants, and at subsequent levels of animal food chains [8]. The production capacities of a thallium recovery installation in zinc smelters are used to a small extent, so the production of thallium can easily be increased as required [5]. Components, such as Ge, Te, and Tl, in e-waste include printed circuit boards ( PCBs), cathode ray tubes (CRTs), liquid crystal display (LCD) screens, light-emitting diode (LED) lights, batteries, circuit boards, and solar and photovoltaic cells (PV) [9]. Each of the above-mentioned wastes has a heterogeneous material composition (organic materials, metals, glass fiber, and ceramic) and the management of recycling these precious metals and hazardous metals requires sophisticated technologies and a multidisciplinary approach [10].

Each year, approximately 20–50 million tons of electrical and electronic equipment waste (e-waste) are produced globally, and this amount is estimated to increase by 3–5% annually [11]. Metals in PCBs consist of a large number of base metals; rare metals, such as Ta, Ga, and other precious metals. Hazardous metals, such as Cr, Pb, Be, Hg, Cd, Zn, and Ni are also present [10]. However, when considering the possibility of recycling these metals, millions of tons of waste of electronic equipment are in circulation and should be taken into account. Recycling and unitary processes of disassembly, separation, grinding, and milling can be the source of uncontrolled emissions of metals into water, soil, and air.

Even with low TCE contents in electrical equipment, the problem becomes critical due to a large amount of produced e-wastes. These amounts also become so large that recovery of these metals from waste becomes feasible and profitable.

In addition, application of these processes cause the migration of TCE metals to nearby environments and/or exposes workers to the harmful effects of metals through inhalation, skin contact, or ingestion [11]. In particular, thallium is a highly toxic metal, listed by the European Water Framework Directive [1] and the United States Environmental Protection Agency (USEPA 2015) as a priority pollutant, of which penetration into surface environments, and its dispersion in soils, sediments, and waters, can occur relatively easily due to the high volatility and solubility of thallium compounds [12]. The characteristics of germanium, tellurium, and thallium, along with the influence of these metals on living organisms, are presented in Table 1.

The Ge content varies in various US soils, from <0.1 to 2.1 mg kg^−1^, and from <1 to 95 mg kg^−1^ in the topsoils of Sweden. The occurrence of Te on Earth is about 1 µg kg^−1^, and its content in rocks ranges from 1 to 5 µg kg^−1^. Tl content ranges from 0.01 to 2.8 mg kg^−1^ and is increased in organic soils [12]. As mentioned earlier, the indicated critical metals, due to their low content in e-waste, are poorly described in the literature. It is difficult to compare the above values with their content in secondary raw materials, as there is no comprehensive information on TCE content per ton of waste.

PCBs are diverse and complex in terms of type, size, shape, components, and composition. In addition, as technology progresses, PCB compositions are continuously changing, making it more difficult to obtain stable material compositions. The presence of plastics, ceramics, and metals in PCBs, in a complex manner, leads to great difficulty in liberation and separation of each fraction. Metals in PCBs consist of a large number of base and rare metals, as well ashazardous metals (such as Ge, Te, Tl) [13,14,15].

At present, there are no literature reports on what the levels of these metals in particular types of e-waste are, as well as no detailed information on recovery processes of the development of Ge, Tl, and Te. The situation is similar in the case of individual WEEE types, e.g., LEDs, as rare earths are also not currently recycled from these devices [16]. The main obstacle in carrying out processes for recycling germanium, tellurium, and thallium are the small amounts of TCE materials that are dispersed in shredded waste. However, due to their toxicity and effect on human health, they require at least proper landfill storage.

Therefore, the shredding of LED backlit displays could have the same effect as that of displays containing mercury-based backlighting systems, i.e., they could lead to hazardous contamination by recyclable fractions. Current EU WEEE legislation does not yet address specific treatments for LED-based products, mainly because the massive use of LEDs in EEE only began recently and there is still little research regarding their potential toxicity and their proper EoL treatment (end-of-life) [17].

The life span of currently produced solar modules is 25–30 years, and, after that, they will require proper management. If they are deposited in landfills, the metals they contain can potentially be released into the environment. PV modules can be stored in ordinary landfills, as long as the contained CdTe does not leach out. In other cases, when the concentration of metals exceeds the limit values (the modules have the ability to release, among others, cadmium), it is necessary to subject them to the recycling processrd or depositing them in a hazardous-waste landfill [18,19]. PV recycling processes begin with the physical separation of individual elements; the modules are then crushed, and the metals are removed in subsequent stages of chemical dissolution, mechanical separation, as well as precipitation or electrolytic deposition. Finally, glass and the metal fractions are recovered (e.g., 80–96% Te, Se, and Pb). Other metals (e.g., Cd, Te, Sn, Ni, Al, and Cu) are contained in sludge, which is then subjected to further recycling processes [7].

Mechanical processing of e-waste, shredded into pieces using hammer mills, is an integrated part of its recycling process. Mechanical process and physical separation techniques (screening, magnetic, eddy current, and density separation techniques) allow to separate non-metals fraction (polymers, ceramics, and glass) from metals and concentrate them in one fraction [20,21,22]. This approach allows maximum material recovery, increases the efficiency of their processing and recovery. The size of the material particle, obtained in the milling process, is one of the most important parameters affecting metal dissolution efficiency during hydrometallurgical e-waste processing [21,22]. However, the individual processes of crushing and milling of e-waste create conditions in which metals may be released if grinding of the products is performed. In addition, the activities of collecting and transporting the fragmented fractions can lead to the dispersion and migration of pollutants in the form of dust containing these metals and other organic compounds into water and soil environments [23]. The process of releasing metals from waste and their migration to the environment is affected by physico-chemical environmental factors. This process is favored by lowering the pH of the environment in which the waste is collected/stored, temperature changes, oxidation and reduction conditions, as well as the processes of material fragmentation and increases in organometallic organic complexity [24,25,26].

Recycling of TCE, including gallium, germanium, indium, or rare earth elements (REEs), is difficult due to their low concentration, dispersion in electronic components (a multitude of products with different concentrations), and material heterogeneity, where metals are combined with alloys, ceramics, glass, composites or tightly packed in material structures [27,28]. Due to these factors, elements may already be lost at the stage of WEEE shredding, which tends to accumulate the fractions of dust and ferrous metals [29].

Worldwide, about 30% of the total germanium consumed is produced from recycled materials, electronic devices, and optical fibers. During the manufacturing of most optical devices, more than 60% of germanium is routinely recycled as new scrap [6]. The basic procedure of obtaining germanium from wastes is leaching using hot sulfuric acid. Among the techniques of separating Ge from other elements (contained in leachate), precipitation, adsorption, solvent extraction, adsorption on chelating, ion-exchange extraction, and vacuum reduction metallurgical process are used [28]. For traditional metallurgical and chemical uses, there were little or no old scrap from which to extract secondary tellurium because these uses of tellurium were highly dispersive. A very small amount of tellurium has been recovered from scrapped selenium–tellurium photoreceptors employed in older plain-paper copiers. Tellurium recycling from CdTe solar cells is still slight, as most CdTe solar cells are relatively new and have not yet reached the end of their use [6]. 

In the article, elements and electronic subassemblies that are carriers of Ge, Te, and Tl, were subjected to disassembly and mechanical treatment (cutting, grinding), and then quantitative analyses were made. Due to the low concentrations of Ge, Te, and Tl in the e-waste stream, materials for testing were selected according to their content. Grinding of the selected metal-bearing electronic elements enabled the release of Ge, Te, and Tl from one non-complicated component matrix (e.g., glass, ceramics only) ensuring accurate determination of the concentration of these metals in a given fraction without major losses. Currently, knowledge on the presence of Ge, Te, and Tl in the electronic scrap circulation is negligible.

This research aimed to determine the distribution of Ge, Te, and Tl in the groundmass of waste in individual fractions and to determine the possible tendency to accumulate/concentrate metals in a particular fraction. In addition to the analyses of Ge, Te, and Tl contents, this article also draws attention to other metals (Ba, Co, Mn, Cu, Ni, and Zn) occurring in the matrices of electronic components, especially harmful elements (Cd, Cr, and Pb). The distribution of metals in the ground fractions of e-waste is crucial knowledge in the analysis of migration issues of pollutants from e-waste collection and recycling plants in the form of dust to the environment. To better understand the relationship between TCE migration to the environment and their mobility in soils in e-waste recycling areas, correlation studies will be discussed in future works.

Our research results evidence the impact of an electrowaste processing plant on an increase of germanium and tellurium concentration in soils in the area directly surrounding the WEEE plant. The direction and extent of emission of dusts (created during mechanical disassembly and shredding) depended on the strength and dominant wind direction. Our results showed also that the WEEE plant had little impact on the increase in thallium concentrations in the topsoil surrounding the plant. The studies showed that the slight increase in the thallium content in the topsoil did not have a geogenic basis, but was only due to the influence of human activity.

## 3. Materials and Methods

### 3.1. Sample Collection and Preparation

For quantitative and qualitative composition tests, elements of electronic waste and electronic components that were carriers of Ge, Te, and Tl were selected; outdoor solar lamps, solar cell, LED TV screen matrix, LCD screens of used mobile phones, as well as uniform electronic elements, such as photoresistors, photodiodes, and phototransistors. Except for TV screen matrixes and LCD screens, all elements were new, and were purchased from an electrical and electronic parts warehouse. LCD screens and the Samsung TV screen matrix came from used electronic equipment, obtained from a local e-waste market. These elements are examples of typical electronic waste that wind up at collection and processing places. Specifications on the e-waste samples used in this study are presented in Table 2.

Elements and sub-assemblies were subjected to deep, manual disassembly, removing all elements that were material ballast and/or covering carriers of tested metals. Plastic housings, covers (solar lamps), polymetric frames protecting the LCD/LED screen structure (mobile and TV screens), rubber or silicone layers (solar panels), and connection pins, (photoresistors, photodiodes) were removed. Only the Ge, Te, and Tl metals carriers were intended for further analyses. The separated material was crushed and cut into smaller size fractions (1 × 1 cm^2^), if needed, and then ground using a knife mill (Chemland, model FW135, Stargard Szczecinski, Poland) to provide an effective liberation of the metals. The milling time was 2 min or more, depending on the type of ground material. Samples consisting mainly of silica were rapidly disintegrated, while ceramic type materials required a longer disintegration time. Magnetic separation was used for the recovery of ferromagnetic metals from residues in ground material (e.g., photoresistors). To determine the degree of metal dispersion in the ground materials, and to obtain the knowledge necessary to determine the degree of release of Te, Ge, and Tl, depending on the grain size of the ground waste, the materials were subjected to grain classification. Samples were sieved in an electromagnetic sieve shaker (Sieve shaker LPzE-2e, Multiserw-Morek, Brzeznica, Poland) equipped with sieves of standard sizes: 1.0, 0.5, 0.2, and 0.1 mm.

The adopted grain range of ground elements (>1.0–<0.1 mm) corresponded to the possible size of dust particles formed during mechanical processes of e-waste scrap treatment. The distribution of individual grain fractions in ground materials is presented in Table 3.

The results presented above allow the conclusion that the smallest fraction, below 0.1 mm, also has the smallest share in the total fraction distribution. On the other hand, in the case of larger fractions, the distribution was unevenly distributed between individual fractions. The exceptions were photoresistors and LCD screens, where the thickest fraction >1.0 was a small percentage of the total. The relationship between the metal concentration vs. size and their distributions is described in more detail in Section 4.3. 

### 3.2. Digestion Method of Electronic Components

The analysis of the content of elements in e-waste material is difficult due to the heterogeneity of the individual components of this waste. There is still no single standardized method and different digestion protocols have been adopted for sample preparation. The lack of a standardized method of analysis has caused discrepancies in the results of various authors and makes it difficult to compare the efficiency of recovery processes [32,33].

The digestion process carried out in an open vessel under reflux conditions, or in a closed vessel assisted by microwave radiation, involving the use of HNO_3_, HCl, HBF_4_, HF H_2_O_2,_ and their mixtures, and are described in many publications [19,34,35,36,37,38]. The efficiency of a digestion protocol for solid waste is dependent on the waste matrix, the chemical form of the metals in the waste matrix, and the acids used in the digestion process [33]. The authors of [32] tested 11 WEEE digestion protocols using various combinations of inorganic acids. It was shown that dissolution of basic metals. Such as Cu, Fe, Ni, Zn, Pb, Al, and the noble metals, Pd, Au, and Ag, from an e-waste matrix with a mixture of aqua regia and HF was found to be the most effective combination for maximum metal extraction. However, using other possible acid combinations, the authors achieved an equally high transition efficiency for metals such as Zn, Pd, Cu, and Ag for solutions, or even better transition for Fe and Sn metals.It isdifficult to standardize the digestion protocol due to the heterogeneity of material in e-waste [39].

There is no available certified reference material for electronic waste or its individual components, such as PCBs or LCD. However, first attempts have been made to develop reference materials (RM) for PCBs, indicating the leaching procedure in diluted aqua regia (AR 50% v/v) and microwave radiation as adequate for determining Ag, Au, Cr, Fe, Sb, Sn, and Zn [33]. Both the results for RM and the results of the analyses of the tested digestions of e-waste protocols refer only to basic and noble metals; there is no analysis for critical metals, such as Ge, Te, and Tl. Lack of certified reference materials (CRMs) for electronic waste, combined with the complex composition of WEEE and the difficulties of sample leaching, has made analyzing this material challenging [33]. As the methodology for the analysis of Cu and other basic metals used by the authors has already been repeatedly verified, the validation of the digestion method has been limited only to metals from the TCE group (Ge, Te and Tl). 

Due to the lack of available CRM for Ge, Te, and Tl in WEEE, CRM NCSDC 73322 (China National Analysis Center for Tron and Steel, Beijing, China) soil was used to verify the adopted methodology. A total of 0.5 g of soil sample was digested in hot aqua regia for 3 h. The results presented in Table 4 confirm that the applied method was appropriate for thallium and tellurium analysis and the results of their digestion were consistent with the levels presented for the certified reference material used in the tests. Germanium can form volatile chloride that is not fully retained by aqua regia [40].

In the digestion process of electronic components, mixtures of HCl and HNO_3_ acids (aqua regia) were used. Aqua regia, as a common medium for digestion of multi-material samples, has been widely used for e-waste [36,41,42]. The ground elements of e-waste were sieved and sorted into five groups: (i) particle sizes larger than 1 mm; (ii) particles sizes between 1 and 0.5 mm; (iii) particles sizes between 0.5 and 0.2 mm; (iv) particles sizes between 0.2 and 0.1 mm; and (v) particles sizes less than 0.1 mm. To determine the metal content of the fractionated material, 0.5 g of each sample was digested using 0.03 L of HCl (35% m/v) and 0.01 L of HNO_3_ (69% m/v) acids. The digestion process was carried out in open systems by heating on a hot plate (95°C ± 5 °C) for 3 h, using freshly prepared aqua regia. All reagents were of analytical grade (APM Poland S.A.-POCH ™ brand from Avantor ™, Gliwice, Poland). The mixture was then filtered through filter paper into a 0.05 L standard volumetric flask. 

### 3.3. Determination of the Total Element Contents 

The total content of Ge, Te, and Tl, as well as other metals and metalloids, were determined using an ICP-MS and ICP-OES spectrometer. The Elan 6100 DRC-e ICP-MS spectrometer (Perkin Elmer, Waltham, MA, USA) was used for quantitative analyses of total Ge, Tl, Te, Cd, Ba, Co, and Mn, while metals, such as Cr, Cu, Ni, Pb, Sr, and Zn were quantified using an Avio 200 ICP-OES (Perkin Elmer, Waltham, MA, USA) spectrometer. 

The ICP-MS apparatus was equipped with a standard ICP quartz torch, cross-flow nebulizer, and nickel cones. Samples and standards were delivered with a peristaltic pump. The spectrometer was optimized daily with a 10-µg/L solution (Mg, Cu, Rh, Cd, In, Ba, Ce, Pb, and U) in 1% HNO_3_ Elan 6100 Setup/ Stab/Masscal solution (Perkin Elmer, Waltham, MA, USA). The concentrations of metals were measured with an internal ^103^Rh standard. 

Germanium determination using ICP-MS spectroscopy is difficult due to numerous spectral interferences: ^70^Ge (^35^Cl^17^O^18^O^+^, ^36^Ar^34^S^+^, ^38^Ar^32^S^+^, ^70^Zn^+^), ^72^Ge (^36^Ar_2_^+^, ^56^Fe^16^O^+^,^40^Ar^32^S^+^, ^40^Ar^16^O_2_^+^, ^55^Mn^16^OH^+^), ^74^Ge (^40^Ar^34^S^+^, ^36^Ar^38^Ar^+^, ^40^Ar^36^S^+^, ^37^Cl_2_^+^,^74^Se^+^) [39]. 

To eliminate these polyatomic interferences, DRC technology with CH_4_ as the reaction gas was used. The flow rate of the reaction gas and the value of the rejection parameter (Rpq) were the key parameters for DRC operation. The optimized CH_4_ flow rate was selected to be 0.4 mL/min and when the Rpq value was 0.65, the signal of ^74^Ge tended to be stable. Under the optimized conditions, the analytical performance of the proposed method was evaluated. Standard solutions with Ge concentrations from 1 to 25 µg/L were analyzed to construct a calibration curve with a correlation coefficient of 0.9998. Limits of detection (LOD) was determined as a three-time value of standard deviation for the blank sample, which was an acidified water sample, used to prepare calibration solution and the dilution of the real samples. The limits of quantification (LOQ) were expressed as three times the value of the limit of detection. The limits of detection and quantitation were 0.05 µg/L and 0.3 µg/L, respectively. 

Direct determination of tellurium in environmental samples by using coupled plasma mass spectrometry (ICP-MS) is often complicated by its low abundance, poor analytical sensitivity, and the presence of xenon interference. Therefore ^125^Te, ^126^Te, ^128^Te, ^130^Te isotopes were measured using correction equations (−0.003404 × ^129^Xe) for ^126^Te, (–0.072617 × ^129^Xe) for ^128^Te, (−0.009437 × ^137^Ba − 0.154312 × ^129^Xe) for ^130^Te. The best results were obtained with the ^126^Te isotope, as was done in the work of Filella and Rodushkin [40]. Standard solutions with Te concentrations from 1 to 25 µg/L were analyzed to construct a calibration curve with a correlation coefficient of 0.9997. The limit of quantitation (LOQ, three times to the LOD) was 0.42 µg/L, while the LOD was 0.14 µg/L. Methodology for determination of the ionic Te(VI) and Te(IV) forms has been described in detail in the work by Grygoyc and Jabłonska-Czapla [43].

Other elements, such as ^114^Cd, ^130^Ba, ^59^Co, ^55^Mn, and ^205^Tl, were measured using an ICP-MS spectrometer in standard mode. The research into the total Cr, Cu, Ni, Pb, Sr, and Zn contents was conducted using an Avio 200 inductively coupled plasma-optical emission spectrometer (ICP-OES, Perkin Elmer, Waltham, MA, USA) with an axial viewing configuration. The operating conditions of the instrument are shown in Table 5. The ICP OES was equipped with a standard ICP quartz torch with a corundum injector tube, a cross-flow nebulizer, and a Scott’s spray chamber. The instrument performance was checked by measuring an optimization solution (Optima Family Multi-Element Standard, Perkin Elmer, Waltham, MA, USA). The calibration mixed standard solutions were prepared from the stock standard solution of 1000 μg/L. The following spectral lines have were for analyses: Cr—205.560 nm, Cu—327.339nm, Ni—221.648 nm, Pb—217.000 nm, Sr—407.771 nm, and Zn—213.857 nm.

### 3.4. Scanning Electron Microscopy (SEM-EDS) and X-Ray Diffraction (XRD)

Observations of the surface of the tested samples were made with a Hitachi S-4200 scanning electron microscope (Mannheim, Germany) using a secondary electron detectors (SE). Chemical composition tests were performed using an X-ray energy dispersion spectrometer (EDS) from Thermo Noran (System Seven, Mannheim, Germany) at an accelerating voltage for the electron beam of 15 keV. Results of EDS microanalyses of the chemical composition included also surface analyses.

The phase composition was analyzed by X-ray diffraction (XRD) using a Panalytical Empyrean diffractometer (Malvern, UK) equipped with PIXCel detector ( and a Johansson monochromator. Data collection was performed over a 2q range of 10–80° with a 0.02°/step using Ni-filtered and Cu radiation.

## 4. Results and Discussion

### 4.1. Total Metal Contents

**Figure 1:**
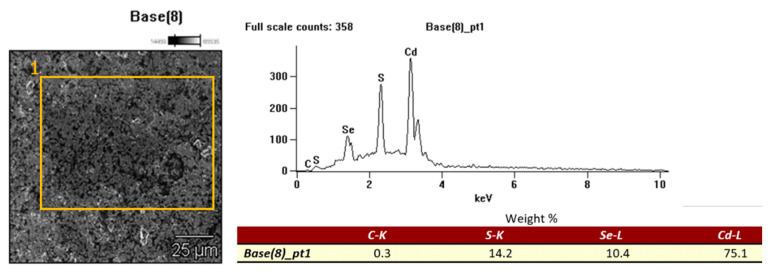
SEM image and EDS microanalysis of photoresistor surface.

The contents of Ge, Te, and Tl, and other metals (Cd, Ba, Co, Mn, Cr, Cu, Ni, Pb, Sr, and Zn) in the analyzed elements and electronic components are shown in Table 6. The content of Ge, Te, and Tl differed depending on the type of electronic component. The highest content of thallium was found in the TV screen material and solar cells in the form of solar panels: 0.81 mg·kg^−1^ and 0.38 mg·kg^−1^, respectively. The main carriers of germanium were photoresistors (2.59 mg·kg^−1^), while tellurium was the material for the LCD screens of mobile phones (0.17 mg·kg^−1^). Among the other metals, copper (6.4–0.6%), chromium (0.5–0.01%), and nickel (0.01–0.008%) were the dominant ones. The presented content characterized the composition of electronic components, in which metalloids of germanium and tellurium (or their compounds) w widely used in electronics for the fabrication of thin-film transistors (TFTs), glasses, optical storage, optoelectronics, or semiconductors [44,45]. Ge, Te, and Tl belong to micro or trace constituents, of which the content in electronic equipment does not exceed <0.1%. For example, the content of germanium in a typical personal computer is 0.0016% of the total weight [46]. However, hazardous components (metals), even in the smallest amounts require specific attention. Contamination with metals is considered a major environmental issue and threat to human health. Among the many metals, Tl, Hg, Cr (mainly hexavalent chromium) Cd, and Pb are the most harmful, are very hazardous to humans, bio-accumulate and have carcinogenic properties [35,36,37]. The maximum values of the concentration of these harmful substances in EEE (electrical and electronic equipment) are clearly defined in the EU Restriction of Hazardous Substances RoHS Directive [47], in which the main goal is to limit the use of certain hazardous substances in electrical and electronic equipment. The RoHS specifies maximum levels for six restricted materials, and among them, EEE products must not contain more than: Pb: <1000 mg·kg^−1^, Cd: <100 mg·kg^−1^, Cr VI: <1000 mg·kg^−1^, Hg <1000 mg·kg^−1^. There are exceptions to the RoHS Directive when it is not possible to replace a given element (substance), or it is not possible to reduce its concentration to the maximum concentration level, without losing the desired physical or physicochemical parameters. These exceptions are listed in the list of ANNEX III [48], among which cadmium and its compounds can be used in electrical contacts. For the case of using cadmium in photoresistors, the limits are expired on 31 December 2013. However, our results of metal content in the tested photoresistors material indicate a significant share of Cd (2313.87 mg·kg^−1^), occurring in tracks as CdS or CdSe. SEM-EDS analysis confirmed the presence of cadmium in the track of photoresistors made with CdS (Figure 1).

### 4.2. XRD Analysis

**Figure 2:**
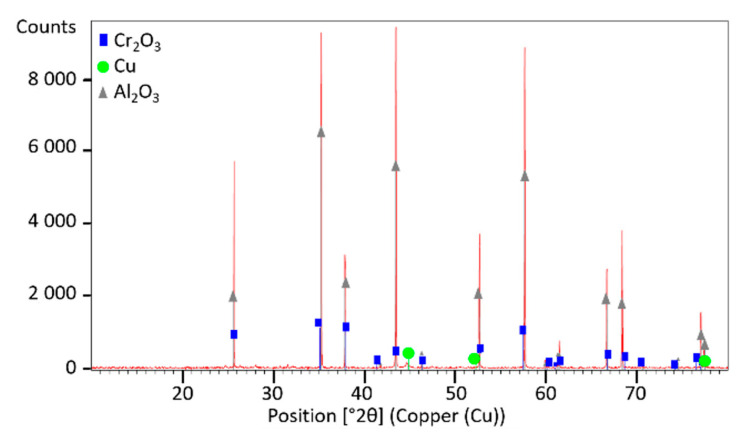
XRD analysis of 0.2–0.1 mm size fraction of photoresistors.

**Figure 3:**
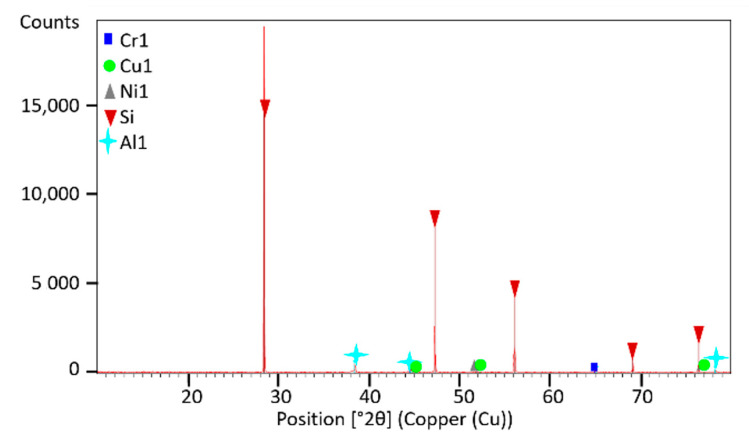
XRD analysis of 0.2–0.1 mm size fraction of solar cell.

**Figure 4:**
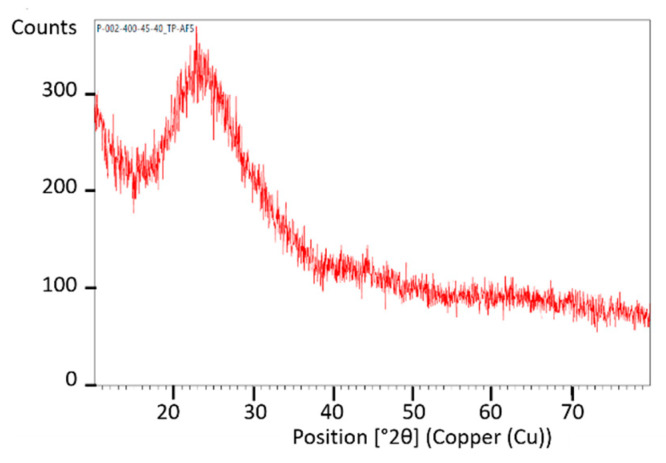
XRD patterns of LCD screen.

Fragmented sample material with a 0.2–0.1 mm size fraction was provided for analyses and the presence of dominant phases in it was characterized using X-ray diffraction (XRD) analyses. The XRD patterns of photoresistors and solar cells are shown in Figure 2 and Figure 3, respectively. XRD spectrum indicates that there are diffraction peaks of alumina (Al_2_O_3_), chromium oxide (Cr_2_O_3_), and copper (Cu) in the material of photoresistors, and of quartz (SiO_2_) in solar cells. No diffraction peaks of other components containing Ga, Ge, and Tl were observed due to their low contents. The observation of other XRD patterns of the e-waste elements indicated the presence of amorphous, glassy, or disordered nanocrystalline material in the sample. Figure 4 shows XRD spectra of the amorphous phase in the sample of the LCD screen. No specific diffraction peaks were exhibited, which is typical for LCD glass containing mainly amorphous silicon [49], minor additives of indium, tin [50], and other elements, as presented in Table 7.

### 4.3. Distribution of TCEs in Different Particle Size

**Figure 5:**
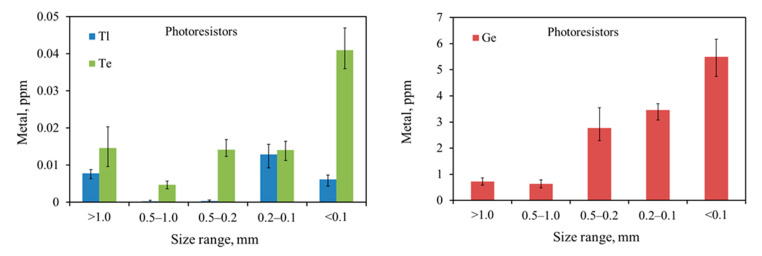

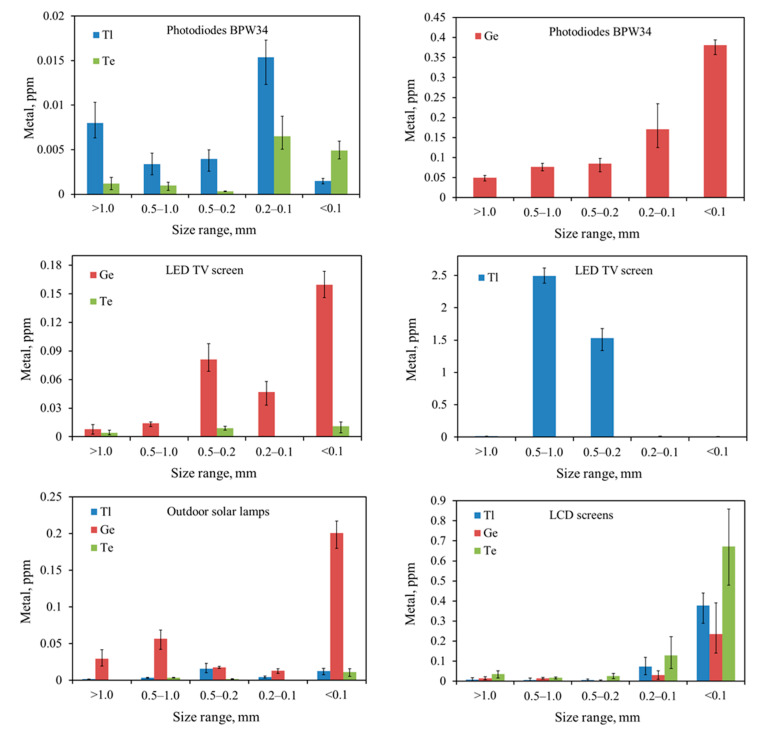
Ge, Te, and Tl content in different particle sizes of ground e-waste.

The Ge, Te, and Tl contents in grains of different size fractions after milling are shown in Figure 5. Due to differences in the content range of the tested materials, the relevant graphs are presented separately. Tl, Ge, and Te were concentrated mainly in the finest fractions. The results of comminution and classification clearly showed that germanium content was the highest in the smallest fraction, <0.1 mm, for all types of examined elements. The distribution of the other two metals (Tl and Te) in the grain classes was varied. However, also, in this case, these metals accumulated primarily in the finest fraction 0.2–0.1 mm and <0.1 mm. Among other metals, cadmium, chromium, cobalt, and manganese were concentrated only in the finest material fraction (<0.1mm) for all ground types of electronics equipment subassemblies.

The results of the content of elements, which are noted for their potential toxicity, in individual grain classes (for selected WEEE elements), are presented in Table 7.

Shredding and grinding WEEE material is an essential step in the recycling chain. This stage ensures the proper release and separation of useful components. Metals can accumulate in the finest fractions, which already enables the separation of economically significant metals at the stage of mechanical processing. Particle separation behavior, based on their size, shape, and density was studied by Bileasan et al. [51]. They concluded that most of the gold (73 wt.%), palladium (66 wt.%), and silver (33 wt.%) were acquired in the finest fraction (<75 µm). A similar observation is presented by the authors of [52,53], who indicated that PCB particles have a heterogeneous shape and are released when they are crushed to a size less than 3 mm; when the particle size decreases, the degree of release of metals from PCBs increases. This tendency was confirmed by the results presented in the above-mentioned article. The observation of the accumulation of metals in the finest fractions is valuable information; on the one hand, in the recycling process during a simple sieving operation, a higher concentration of metals can be obtained, and on the other hand, metals concentrated in a fine fraction can easily migrate to the environment during uncontrolled e-waste treatment processes.

The contribution of the most harmful elements (Cd, Cr, ad Tl) is particularly dangerous due to the possibility of their easy release into the environment, transport over long distances, or exposing workers to the harmful effects of metals entering their bodies through inhalation, contact with the skin, or ingestion [11]. The content of other metals (Ba, Co, Cu, Mn, Ni, Sr, and Zn) is diverse at different fractions. The Cu content was the highest, mainly in 1–0.5 mm, while Ni, Sr, and Zn were concentrated in the finest fractions obtained in the sieving process.

## 5. Scope for Future Work 

TCEs are undergoing significant changes in terms of the cycle at the Earth’s surface due to their increasing use in a variety of technological applications. Environmental contamination by e-waste recycling is an emerging global issue. E-waste collection plants often carry out preliminary disassembly of electrical and electronic equipment; this also includes the organization of stations or automatic lines enabling the processing of WEEE. Even though the plants use good practices of WEEE and the application of high processing standards, even temporary collection of e-waste or their processing (separation) by grinding can be a source of uncontrolled emissions of pollutants into water, soil, and air in the form of dust emissions containing metals and other organic and non-organic compounds. Therefore, for a complete picture of how Ge, Tl, and Te migrate to soil, as well as their nature and the emission range around e-waste recycling plants, full material analyses of TCE-bearing e-waste components is necessary. In our future research we will present results relate to influence of source of TCE emission from WEEE plants on the environment. It will be particularly important to study the correlation between magnetic parameters and the content of elements, such as Ge, Te, Tl, Cd, Co, Fe, Ni, and Zn, in soil.

## 6. Conclusions

Ge, Te, and Tl are little known elements, the use of which, as components of electronics in new technologies, grows every year. It is necessary to comprehensively analyze the issues of WEEE material characterization in relation to elements, such as Ge, Te, and Tl, as well as an analysis of the methods of their treatment, recovery, the mechanisms of their release from WEEE, carried as dust to the environment, in addition to the environmental and human impacts. Our research is one of the first to pay attention to the problems of rare elements, and to identify the quantitative and qualitative composition of electronic elements containing Ge, Te, and Tl and their grain size distribution of the ground material.

The results of quantitative and qualitative research on electronic components and sub-assemblies containing Ge, Te, and Tl revealed that the contents of metals increase with decreasing particle size. Ge, Te, and Tl are concentrated in the finest fractions 0.2–0.1 mm and <0.1 mm of ground e-waste. In addition to these, other harmful elements, such as cadmium and chromium, were concentrated in the finest material fraction (<0.1 mm) as well. Toxic metals in the finest fractions might be easily released into the environment during mechanical processing of e-waste, which is particularly dangerous. The contents of other metals (Ba, Co, Cu, Mn, Ni, Sr, and Zn) in studied e-waste are diverse in sieve fractions. Due to the lack of CRM for Ge, Te, and Tl in WEEE, the correctness of the digestion method, using aqua regia, was verified against the CRM for soil. However, during the adopting of this methodology, the possible losses of Ge should be taken into account and the application of a different system and medium (Ge digestion) should be considered.

The obtained results will be used for further investigations on the anthropogenic migration of Ge, Te, and Tl to the environment in areas associated with e-waste recycling and to determine the mobility of speciation forms of metals in the soil.

## Figures and Tables

**Table 1:** Characteristics of TCE metals with the influence of these metals on living organisms.

Metal	General Description	Impact	Ref
Germanium	widely distributed in the body and its accumulation and retention in the organs depending on the chemical form of germanium. no definite evidence for the mutagenicity of germanium but under certain conditions it inhibits the mutagenic activity of other substances.	causes toxic effects in humans. organic compounds destroy cancer cells and reveal antineoplastic properties in human. inorganic forms (especially when inhaled) may be toxic in high doses.	[6,13,14]
Thallium	very toxic. its salts are considered to be the most toxic compounds known. toxicity of Tl has not been greatly studied, but its harmful impact has been observed on both humans and animals.	contents of Tl in most mammalian tissues <200 µg kg^−1^. increased level, at 500 µg kg^–1^ (in the skin) is easily absorbed through the skin. ingested and inhaled Tl is also harmful to organisms. general exposure has been observed in the population living in the vicinity of a cement plant (discharges high-Tl containing dust). other Tl sources: coal combustion, roasting of sulfide concentrates, and manufacturing of Tl-containing products.	[6,14]
Tellurium	has no biological role. all its compounds are highly toxic. considered to be a teratogenic agent, however, acute poisoning is rare.	workers exposed to 0.5 to <10 µg Te m^–3^ have a garlic-like odor (a symptom of the Te intoxication). permissible exposure limits for Te in inhaled air vary for different compounds, from 0.1 mg m^–3^ for elemental and dioxide Te, to 10 mg m^–3^ for telluride compounds.	[14]

**Table 2:** Specifications of e-waste samples used in the study.

Type of E-Waste with a Ground View	Characterization and Metal Contents
Photoresistors Light Dependent Resistors (LDR) 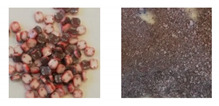	Made of two electrodes: PbS, PbSe, InSb, and most commonly CdS and CdSe (now restricted in EU because of environmental issues with the use of Cd) and ceramic covering undoped materials, such as silicon or germanium [29]. For visible light, photoresistors are made of silicon, PbS, PbTe, and infrared, InSb, PbSnTe, and HgCdTe.
Photodiodes BPW34 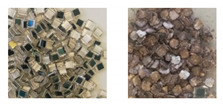	Most often produced from silicon, germanium, GaInAs, PbS, HgCdTe [30].
Phototransistors 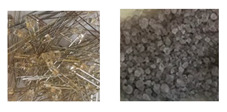	Phototransistors are made up of Group-III and Group-V materials, such as GaAs, in such a way that gallium and arsenic is each used on either side of the transistor.
Outdoor Solar Lamps 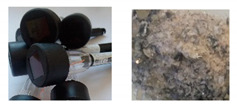	Most often are made out of single crystalline silicon, a semiconductor material. A solar cell has two different layers of silicon. Films with high transmittance and high electrical conductance, such as indium tin oxide [31].
Solar Cell 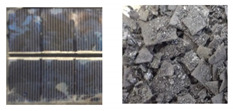	Nowadays predominant PV technology (2nd generation cells) are made from thin-film solar cells, that include amorphous silicon, CdTe and copper, indium, gallium, selenide (CIGS). Germanium is used as semiconductor materials for multi-junction solar cells.
TV LED Screen 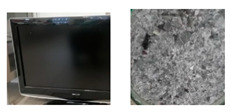	LEDs screen consists of aluminum, indium, gallium phosphide. Germanium is used in glass for fiber-optic cables, infrared optics (night-vision), in semiconductors.
LCD Screen 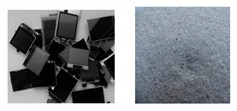	TheLCD contains the thin-film-transistor (TFT) panel, which is relevant for its indium content [16]. More than 80% of indium in the world is produced for indium tin oxide (ITO) coatings used in LCDs [19].

**Table 3:** The distribution of individual grain fractions in the ground material.

Type of Material	Size Fraction, mm				
	>1.0	1.0–0.5	0.5–0.2	0.2–0.1	<0.1
	% Mass				
Photoresistors	5.4	35.0	35.3	14.9	9.4
Photodiodes BPW34	33.6	37.4	13.6	9.3	6.1
Outdoor solar lamps	37.3	22.7	21.7	10.2	8.1
TV LED screen	45.7	22.5	19.0	7.4	5.4
LCD screen	3.4	16.2	44.9	18.2	17.3
Solar cell	23.7	15.8	26.7	23.6	10.2
Phototransistors	24.3	44.1	24.4	4.8	2.4

**Table 4:** Metal contents in soil determined by aqua regia vs recovery of a certified reference material soil (CRM)**.**

NCSDC 73322 Soil			
Element	Concentration, mg·kg^−1^	Certified Value, mg·kg^−1^	Recovery, %
	Aqua Regia		
Te	0.16 ± 0.02	0.16 ± 0.06	100%
Ge	1.45 ± 0.24	1.9 ± 0.3	75%
Tl	0.91 ± 0.05	0.94 ± 0.25	97%

**Table 5:** Operating parameters of the ICP-MS.

Parameter	Value
ICP-MS	
RF power (W)	1125
Plasma gas flow (L/min)	15
Nebulizer gas flow (L/min)	0.76–0.82
Auxiliary gas flow (L/min)	1.15–1.16
Nebulizer type	Cross flow
Plasma torch	Quartz
Scanning mode	Peak hopping
Dwell time (ms)	100
Sweeps/reading	20
Number of replicates	3
Rpq value	0.65
CH_4_ flow (mL/min)	0.4

**Table 6:** Average content of metals in components and sub-assemblies of electronic devices.

Element, mg·kg^−1^	Type of Electronic Equipment Subassembly						
	Photoresistors	Photodiodes BPW34	Outdoor Solar Lamps	TV LED Screen	LCD Screen	Solar Cell	Phototransistors
Te	0.020	0.003	0.003	0.005	0.170	0.030	0.040
Ge	2.590	0.150	0.060	0.06	0.062	0.131	0.231
Tl	0.010	0.010	0.011	0.810	0.091	0.381	0.142
Cd	2313	49.82	7.140	1.96	0.26	1.020	51.88
Ba	104.9	22.73	44.15	5.542	158.8	30.23	33.78
Co	8.691	2.060	1.680	1.561	2.462	8.071	5.110
Mn	195.4	14.53	11.16	9.540	15.62	42.21	44.53
Cr	5738	317.2	137.4	129.5	2875	685.3	400.6
Cu	64,177	12709	160.83	104.55	109.9	2565	2101
Ni	157.4	173.7	87.03	78.27	77.07	313.1	354.6
Pb	7.541	19.44	764.6	25.46	8.111	1766	134.3
Sr	19.68	61.85	18.71	145.5	570.6	74.90	73.17
Zn	169.6	62.74	37.37	25.96	26.30	164.1	38.20

**Table 7:** Harmful elements in a different fraction of ground WEEE.

Fraction, mm	WEEE	Content, mg·kg^−1^		
		Cd	Cr	Pb
>1	Photoresistors	312.16	404.90	5.00
	Solar lamps	0.50	13.59	32.34
1.0–0.5	Photoresistors	45.15	259.40	5.00
	Solar lamps	0.13	5.95	15.14
0.5–0.2	Photoresistors	1354	3770	6.20
	Solar lamps	0.35	13.47	20.74
0.2–0.1	Photoresistors	3243	10130	8.30
	Solar lamps	0.42	18.52	36.55
<0.1	Photoresistors	6615	14125	13.20
	Solar lamps	34.30	635.40	3718

## Data Availability

Not applicable
